# Early Life Exposure To Multiple Metals, Nutrition, and Growth in Children – A Scoping Review

**DOI:** 10.1007/s40572-025-00502-w

**Published:** 2025-10-02

**Authors:** Gauri Desai, Nicholas Blatto, Michelle L. Zafron, Carole Rudra, Katarzyna Kordas

**Affiliations:** 1 Department of Epidemiology and Environmental Health, University at Buffalo, Buffalo, NY USA; 2 University Libraries, University at Buffalo, Buffalo, NY USA

**Keywords:** Metal mixtures, Prenatal growth, Postnatal growth, Nutrients

## Abstract

**Background:**

*In utero* and childhood exposure to toxic metals is associated with poor child growth, a predictor of adverse health outcomes. Most existing research focuses on exposure to single metals; the effects of metal mixtures largely remain understudied. Further, few studies consider how diet/nutrients interact with metal mixtures.

**Objective:**

To synthesize research on the relationship between *in utero* and childhood metal mixture exposures, nutritional status-metal exposure interactions, and child anthropometric outcomes.

**Methods:**

PubMed and Embase were used to search literature published in 2010–2023. Included studies consisted of at least two *in utero* or childhood toxic metal exposures and examined anthropometric parameters as their main outcomes. Included articles underwent full-text screenings. Information on exposures, findings, nutritional variables, and statistical methods was extracted.

**Results:**

After deduplication and title and abstract screening, 95 publications were included; 70 on prenatal growth and 25 on postnatal growth. Nutritional status/diet was assessed as an effect modifier in 4.3% studies on prenatal and 12% studies on postnatal growth. Birthweight (91.4%), and height and body mass index (64%) were common indicators of prenatal and postnatal growth, respectively. Finally, 41.4% of studies on prenatal and 20% on postnatal growth included statistical models that tested for mixture effects.

**Conclusion:**

Although many studies included multiple metals, their mixture effects largely remain untested. Additionally, inclusion of nutritional status/dietary intakes in statistical models is rare, highlighting the need for further research.

**Supplementary Information:**

The online version contains supplementary material available at 10.1007/s40572-025-00502-w.

## Introduction

Linear growth and body size development in early life are critical determinants of health and quality of life [1]. For example, being low birth weight (LBW) or small for gestational age (SGA) has been associated with higher risks of morbidity and mortality in childhood [2], as well as several chronic diseases in adulthood [3]. Impaired linear growth in childhood is also related to higher risk of developing adverse health outcomes, including kidney dysfunction, higher blood pressure and blood sugar, poor neurocognitive development, and infectious diseases including diarrhea, tuberculosis, and meningitis [4]. At the other end of the spectrum, large birthweight for gestational age (LGA) is related to higher likelihood of overweight and obesity across childhood, type 1 diabetes, hypertension, and metabolic syndrome [5]. Furthermore, larger body size, specifically overweight and obesity, can have profound effects on the physical (sleep apnea, type 2 diabetes, asthma, high cholesterol, insulin resistance, etc.), emotional, and social (teasing, bullying, marginalization, etc.) health of children, as well as academic performance [6].

Several environmental exposures can impact children’s growth potential. Metals and metalloids such as lead, arsenic, cadmium, and mercury have been shown to negatively impact *in utero* and postnatal growth of children [7]. Recent reviews and meta-analyses indicate little evidence of effects of mercury [8] and arsenic [9] exposure on fetal growth. Conversely, early life arsenic exposure was modestly, yet negatively associated with birth weight and early childhood growth [9]. Maternal exposure to cadmium also had a modest association with birth weight but not birth length or head circumference [10]. Between mothers with BLLs >5 µg/dL compared to ≤ 5 µg/dL, birthweight was estimated to differ by ~ 230 g [11]. An additional narrative review has provided further details on the link between the prenatal exposure to arsenic, cadmium, lead, and mercury and pre- and post-natal growth; inverse associations were observed between lead and arsenic exposure and birth weight, lead and cadmium exposure and preterm birth, and arsenic exposure and both birth length and head circumference [12].

To date, the effects of metals on child growth have typically been assessed individually, rather than as mixtures [13]. However, exposures to metal mixtures are more common than single metal exposures [13], and a better understanding of the mixture effects on different aspects of growth across the early life span is needed. A systematic review of 34 studies published in 2024 showed consistent associations between metal mixtures and pregnancy outcomes, including infant size at birth [14]. Similar reviews are not available for postnatal growth metrics. Furthermore, while the relationship between nutritional factors and child growth is established, the impacts of nutritional status or dietary intakes on the associations of metals with prenatal or postnatal growth is poorly understood and has not been previously reviewed.

This scoping review synthesizes existing research with two aims: (1) to identify and describe the current literature on the associations between *in-utero* and postnatal exposures to multiple metals and growth in children from birth to 18 years of age; (2) to describe the extent to which nutritional status or diet is considered in the relationship between metal mixtures and child growth.

## Methods

To address the study aims, we described seven features of the current literature: (1) study designs; (2) timing of exposure; (3) metals included and their exposure levels; (4) study endpoints; (5) statistical models employed for multiple exposures; (6) inclusion of nutritional status or diet as an effect modifier; (7) observed effects. In addition to linear growth (birth length, length/height, upper arm length, length for age, peak height velocity), we considered anthropometric indicators of body weight/fatness (birth weight, weight, body mass index, weight for length, percent body fat, skinfold thickness, proportion of fat mass, peak weight velocity). We selected a scoping review approach as it allows the flexibility of delving into multiple aspects of studies and a broader view of what has been published on a given topic.

We utilized PubMed and Embase and developed a comprehensive search strategy with the assistance of a health sciences librarian. Search terms were harvested using the Yale MeSH Analyzer. Four categories of terms were developed (Supplemental Table 1): children/childhood (population), toxic metals (exposure), growth (outcome). Both database-specific subject terms and keyword terms were used in the strategy. Included studies met the following eligibility criteria: (1) Age of the study population: 18 years or younger; (2) Publication dates: January 1, 2010, to August 7, 2023; (3) Publication language: English only; (4) Study participants: Human (not animal or in vitro studies; 5) Study type: An original research paper on cross-sectional, case-control or cohort study designs (not abstracts, reviews, editorials, and commentaries); 6) Exposure: At least 2 toxic metals measured in blood, urine, teeth, hair, occurring during pregnancy or childhood; 7) Outcome: *in utero* or postnatal growth parameters (birthweight, length, child height, weight, BMI, birth size, arm length, arm circumference, head circumference, femur length, height velocity, weight velocity, skinfold thickness, percent body fat, proportion of fat mass, weight for length, length for age). The publication year was limited to 2010 and onward because multi-metal studies were rare prior to that.

Search results from each database were downloaded into EndNote™ 20. Retracted articles were excluded. To deduplicate, we utilized the deduplication features of EndNote™ 20 and Covidence. Title and abstract screening were conducted in Covidence by two independent reviewers. At this stage, articles were excluded if they did not meet the eligibility criteria listed above. For screening decisions that did not match between reviewers, conflicts were resolved by a third reviewer who examined the title, abstracts, and proposed reasoning for exclusion.

The full text of articles that were deemed eligible were examined by two independent reviewers. At the full-text review, reasons for exclusion were recorded in Covidence as wrong study design, wrong exposure, wrong outcome, not a peer-reviewed paper, and duplicate. Conflicts were again resolved with the help of a third reviewer. Several study features were extracted using a prepared Excel table to address the study aims.

## Results

### Overview

**Fig. 1:**
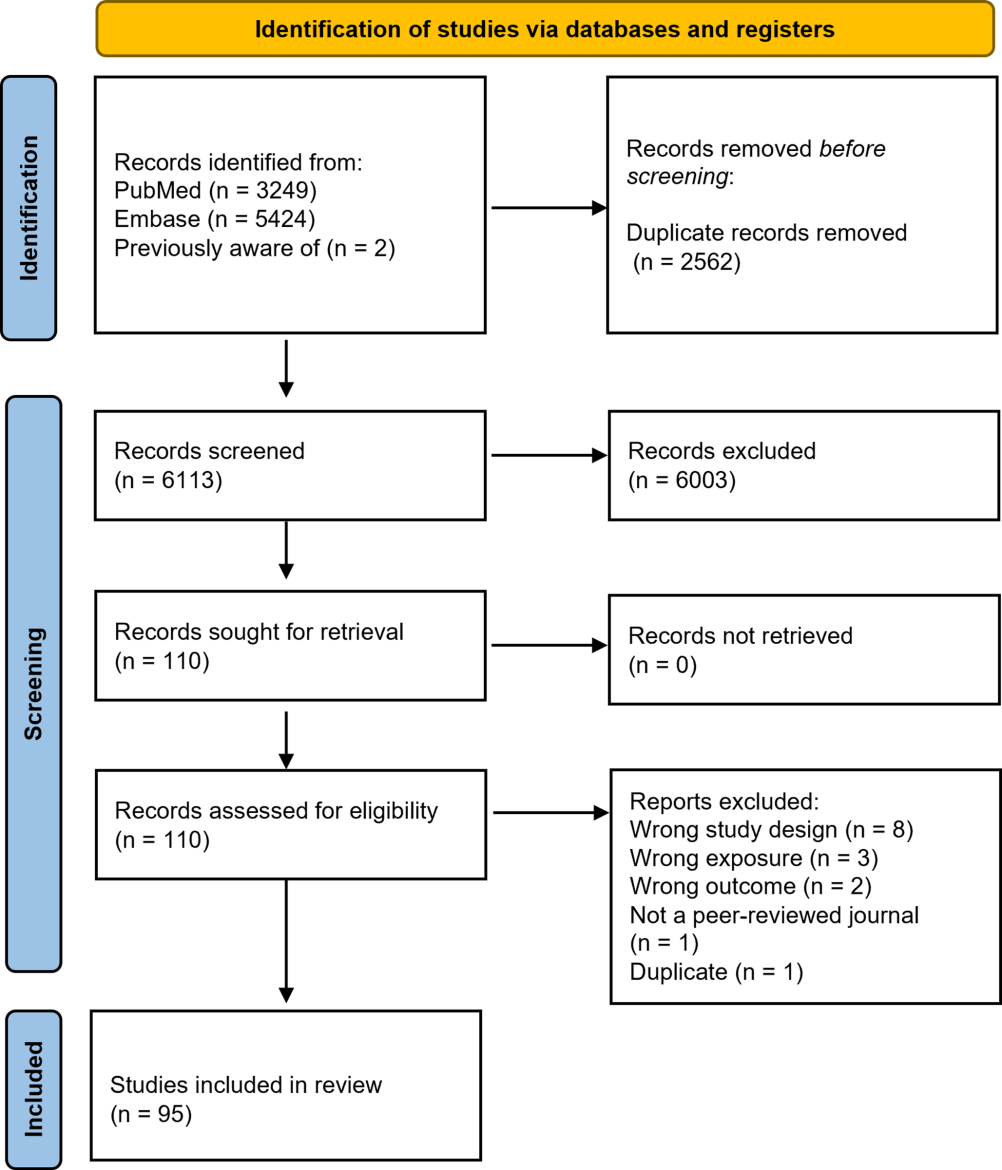
PRISMA flow diagram [15] for the scoping review on metal mixtures and growth

Figure 1 documents the review process and outlines reasons for exclusion at full text review. As shown, 95 publications met the inclusion and exclusion criteria and were ultimately included in the scoping review. Of these, 70 publications referred to growth measured prenatally or immediately after birth and 25 measured growth indicators in the postnatal period. Evidence and study features on pre- and postnatal growth are presented separately. To address the first aim of identifying and describing the current literature on the associations between multiple metals and child growth, study characteristics are presented, followed by a description of study findings and summary of the weight of evidence. For the second aim, the consideration of nutritional status or diet in the relationship between metals and child growth is evaluated.

### Metal Exposures and Prenatal Growth

#### Study Characteristics

Supplemental Table 2 details the characteristics of the 70 studies included in this scoping review that focused on aspects of prenatal growth. Briefly, most of the studies were conducted in the United States (*n* = 17) and China (12), followed by Japan (7) and Spain (4). Lead was measured most frequently (63 publications), followed by cadmium (59), mercury (48), arsenic (44; 4 of these included As speciation) and manganese (39). Selected characteristics are summarized in Table 1. Birth (or estimated fetal) weight was the most common outcome measure, followed by aspects of linear growth (birth length or femur length), head circumference, and finally other circumference (chest, abdomen, or mid-upper arm).

**Table 1:** Characteristics of 70 studies included in the scoping review focused on metal exposures and growth in the prenatal period (*n* = 70)

Characteristic	Number	Frequency
Study year		
2010–2015	11	15.7
2016–2020	31	44.3
2021–2023	28	40.0
Design		
Case-control	3	4.3
Cross-sectional	14	20
Cohort	53	75.7
Growth outcome examined^1^		
Birth or fetal weigth	64	
Birth or femur length	28	
Head circumference	28	
Chest or abdominal circumference	4	
Mid upperarm circumference	2	
Biosample source		
Mother	44	62.9
Infant	10	14.2
Both mother & infant	16	22.9
Maternal sample^1^		
Blood	35	50.0
Urine	9	17.1
Blood & urine	12	12.9
Other^2^	14	20.0
Infant sample^1^		
Cord	22	
Other^3^	6	
Laboratory method^1^		
AAS	13	
ICP-MS	60	
Other exposures measured		
No	55	78.6
Yes	15	21.4
Statistical model to consider multiple metals		
No	26	37.1
Multiple metals in models, including interactions	15	21.4
Mixture or overall effect tested^4^	29	41.4

^1^Multiple response options possible

^2^Category includes hair, placenta, toenails

^3^Category includes baby teeth, hair, meconium, postnatal blood, toenails

^4^Seven studies used models based on principal component anaysis, exploratory factor analysis, cluster analysis, or sum of metals

To determine metal exposures, 62.9% of studies took only a maternal biological sample (predominantly blood). For sampling from the infant, cord blood predominated, but infant teeth, hair, meconium, postnatal blood, and toenails were collected in some studies. Maternal samples were predominantly taken during pregnancy or at delivery, but some studies reported sampling postpartum [16–21]. There was no standard time for sample collection, and 25 studies did not specify timing. Furthermore, some studies collected samples within a narrow window (e.g., 8–12 weeks of gestation), while many others had a much wider window for collection (e.g., 14–28 weeks or 9–40 weeks of gestation). Supplemental Table 3 details the exposure levels for the 15 most reported metals in blood, serum or urine.

With respect to the statistical models employed to test metal-fetal growth associations, 21.4% of studies included mutual adjustment for multiple metals in their models and/or tested statistical interactions between pairs of metals (Table 1). Another 41.4% of studies specifically used mixture models (Bayesian Kernel Machine Regression (BKMR), Weighted Quantile Sum regression (WQS), quantile g-computation or other), or estimated cumulative effects via factor, principal component or cluster analysis, or the sum of metal exposures.

#### Study Findings and Weight of Evidence

Although all studies measured multiple toxic elements, 26 (nearly 40%) of the reviewed studies examined the relationship between individual elements and fetal growth. Of these, most focused on just four elements: lead, cadmium, mercury, and arsenic. Supplemental Table 4 presents the association of commonly studied elements and fetal growth or infant size at birth.

Among the 29 (41.4%) studies that investigated the effects of mixtures, factors integrating multiple element exposures or the sum of exposures, 10 showed negative associations with at least one aspect of growth, three showed mixed effects, seven found null associations, and four did not report on mixture/joint effects (Supplemental Table 4).

#### Inclusion of Nutritional Status and Dietary Intake

Maternal nutritional status is a critical predictor of infant growth. Accordingly, 67.2% of studies on prenatal growth included a measure of BMI (assessed prior to, during or immediately post-pregnancy), while 5.7% of studies measured another aspect of nutritional status (e.g., hemoglobin/anemia, glucose tolerance or another nutrient biomarker in blood) (Table 2). On the other hand, 27.1% of studies did not assess maternal nutritional status. Similarly, 61.4% of studies took no account of maternal diet. Those that did, assessed a wide range of dietary factors, including fish/seafood intake (*n* = 12), prenatal supplement use (*n* = 9), or comprehensive nutrient intake or dietary patterns (*n* = 7). Due to increasing reliance on ICP-MS techniques for exposure assessment, many studies had measures of trace elements that are nutrients (e.g., selenium, magnesium, calcium, potassium, zinc, etc.) and some [22] specifically investigated the associations of trace elements that are nutrients with infant size at birth. Maternal nutritional status, predominantly BMI, was utilized as a covariate in 58 studies, while 8 studies adjusted for other indicators such as maternal hemoglobin or serum lipids (Table 2). Interestingly, only 3 studies investigated the statistical interaction between maternal nutritional status or diet and metal exposure on health outcomes [23–25].

**Table 2:** Consideration of nutritional status or diet in the relationship between toxic element exposure and fetal growth (*n* = 70)

Characteristic	Number	Frequency
Pregnancy nutritional status measured		
No	19	27.1
BMI or height/weight^1^	47	67.2
Other^2^	4	5.7
Any aspect of maternal diet assessed		
No	43	61.4
Yes	27	28.6
Aspects of diet assessed in studies^3^		
Fish or seafood intake	12	
Prenatal supplement intake	9	
Intake of other single foods (e.g., milk, tea)	6	
Comprehensive nutrient intake or diet pattern	7	
Nutritional status included in statistical models^3^		
BMI/height/weight/gestational weight gain	53	
Other^4^	8	
Effect modification by nutritional status or diet tested		
No	67	
Yes	3	

^1^Measured prior to or during pregnancy

^2^Other nutritional status included

^3^Categories not mutually exclusive

^4^Other nutritional status includes maternal hemoglobin or anemia, blood glucose, gestational diabetes/hypertension, serum lpids, creatinine in urine

## Metal Exposures and Postnatal Growth

### Metal Exposures and Postnatal Growth

Table 3 and Supplemental Table 5 summarize key characteristics of the 25 studies conducted in the postnatal period. Many of the studies took place in the US (*n* = 6), followed by China (*n* = 3), and Bangladesh (*n* = 2) and were published between 2021 and 2023 (*n* = 11). The most common metals studied were lead (*n* = 25), cadmium (*n* = 19), arsenic (*n* = 14), mercury (*n* = 12), manganese (*n* = 8), and selenium (*n* = 8). Children’s blood (*n* = 7), urine (*n* = 7), and maternal blood (*n* = 5) were the most common biosamples used to assess exposure levels, and ICP-MS (*n* = 20) was the most common laboratory analysis technique employed for that purpose. Only 5 (20%) studies tested the associations between metal mixtures and child growth; 4 [26–29] of these utilized BKMR and 1 [30] utilized WQS. Of the studies that did not test mixture effects, 13 included multiple metals in statistical models while 7 did not (Table 3). Supplemental Table 6 presents the exposure levels of the 6 most common metals among studies on postnatal growth. Supplemental Table 7 summarizes the associations between 6 commonly studied metals and postnatal outcomes.

**Table 3:** Characteristics of studies that included outcomes assessed in postnatal period (*n* = 25)

Characteristic	Number	Frequency
Study year		
2010–2015	5	20
2016–2020	9	36
2021–2023	11	44
Design		
Case-control	0	0
Cross-sectional	16	64
Cohort	9	36
Biosample source		
Mother	4	16
Child	17	68
Both mother & child	4	16
Maternal sample		
Blood	5	20
Urine	1	4
Breastmilk	1	4
Cord blood	2	8
Child sample^1^		
Urine	7	28
Blood	7	28
Urine + blood	3	12
Fingernails	1	4
Hair	2	8
Cord blood	1	4
Laboratory method^1^		
AAS	4	16
ICP-MS	22	88
HPLC with ICP-MS	2	8
Ion-specified electrode	1	4
Other exposures measured		
No	20	80
Yes	5	20
Growth outcome examined^2^		
Height	16	64
Weight	11	44
BMI	16	64
Waist circumference	4	16
Head circumference	4	16
Other measures^3^	13	52
Statistical model to consider multiple metals		
No	7	28
Multiple metals in models, including interactions	13	52
Mixture or overall effect tested	5	20

Note: ^1^1 study included AAS and ICP-MS, 1 ion specified electrode and ICP-MS, 2 HPLC and ICP-MS. The totals do not add to 100% because of the overlap

^2^ Includes height-for-age Z score, weight-for-age-Z score, and BMI-for-age Z score

^3^Includes birth weight, low birth weight, SGA/LGS, upper arm length, arm circumference, chest circumference, length for age, weight for length, % body fat, skinfold thickness, proportion of fat mass, post birth weight gain, peak weight velocity, peak height velocity

Height and weight were the most common outcomes in the postnatal period, included in 16 studies (standing height/height-for-age z score/length-for-age/weight-for-age z score/BMI/BMI-for-age z score). Five studies included head circumference [26, 31–34] as the outcome, 3 of which [26, 31, 32] were conducted in areas characterized by low levels of exposure. Four studies focused on waist circumference [26, 29, 35, 36], one [35] among a population with high levels of exposure.

### Inclusion of Nutritional and Dietary Components

Dietary intakes were included in 14 studies (Table 4). Of these, several collected data on intakes of foods known to be sources of metal exposure, such as seafood (arsenic and mercury) [26, 27, 31, 37], rice (arsenic) [26, 31], and meat (cadmium, arsenic, mercury) [31, 37], but only 2 included these foods as covariates in statistical models [26, 27]. Data on micronutrient and supplement intake were part of 6 studies [23, 33, 38–41], but only 1 included these in the statistical model [38]. The levels of essential minerals such as selenium, manganese, and zinc were included in several studies, typically as part of a multi-element suite measured via ICP-MS; 15 studies [23, 26, 28–30, 34–36, 38, 42–47] assayed these essential minerals in blood [23, 28–30, 34, 36, 42, 44], urine [26, 35, 45–47], hair [43], or nails [38]. Four studies [26, 28–30] included these measures as part of the exposure mixture. Selenium was the most common essential mineral, assessed most frequently in blood [23, 28–30, 42], followed by urine [45] and fingernails [38]. Tests of interaction of individual metals with essential minerals were not common and were included in only 3 studies [38, 43, 48].

**Table 4:** Consideration of nutritional status or diet in the relationship between toxic element exposure and postnatal growth (*n* = 25)

Characteristic	Number	Frequency
Nutritional status measured		
Maternal prepregnancy/postnatal BMI^1^	8	32
Child micronutrient status	9	36
Other^2^	1	4
None	7	28
Any aspect of child’s diet assessed		
No	11	44
Yes	14	64
Aspects of diet assessed in studies^3^		
Intake of specific foods/food groups	7	28
Micronutrient intake	6	24
Overall diet/diet quality	1	4
Nutritional/dietary variables included in statistical models^4^		
No	11	44
Yes	14	64
Effect modification by nutritional status or diet tested		
No	15	60
Yes	3	12

^1^Includes one study that measured maternal height and weight but not BMI

^2^Maternal micronutrient status measured

^3^Categories not mutually exclusive

^4^Includes maternal/BMI

## Discussion

This scoping review revealed a large body of literature, consisting of 95 studies published up to August 2023 across different settings. The review also showed considerable variability with respect to the biological samples collected, the timing of sample collection, the metals assessed, exposure levels, the outcomes measured, and the statistical models (including covariate adjustment). These study differences also contributed to the large variability in findings, precluding the ability to make clear conclusions on the effects of metals on fetal or child growth beyond lead and cadmium. Although mixture models are increasingly common, they are inconsistent with respect to the specific models used and metals included. Several studies included data on nutritional or dietary components, predominantly individual foods and minerals, but few studies included those data in statistical models. Several recommendations can be made regarding the design of studies investigating the effects of metal mixtures on fetal and child growth.

### Sample Collection

The reviewed studies represented wide variability with respect to which samples were collected and when, likely due to a combination of acceptability by the study population and convenience of collection. Maternal blood and urine and umbilical cord blood were most frequently collected in studies on prenatal growth, and child blood and urine among studies on postnatal growth, but the disparity in the collection periods makes comparison across studies challenging.

Given that blood volume expands in the second trimester of pregnancy potentially affecting the interpretation of blood or serum biomarkers [49], and that there may be trimester-specific windows of susceptibility to the developmental effects of metals [50], designing sampling to fall more discretely within specific periods of pregnancy is highly recommended. Additional issues pertain to the acceptability or utility of certain biomarkers depending on the biological sample in which they are measured. These are further discussed below.

### Metal Assessment

The studies included in this scoping review ranged greatly in the number of metals assessed, from 2 (minimum number to be included in this review) to 21 (median number of metals assessed = 5). Lead, cadmium, mercury and arsenic were most common. In recent years, due to availability of ICP-MS techniques, the number of elements studied has increased considerably, and some studies have begun to report on rare earth metals and minerals, in addition to heavy metals [51]. However, when mixture effects are investigated for more than 3–4 metals, the scientific justification is often lost. Thus, it is unclear why certain metals or trace elements are expected to affect growth and through what mechanisms. An additional consideration is that the matrix in which metals are measured is important. According to a comprehensive review on 12 metal biomarkers (essential and non-essential), not all biological samples are appropriate for the assessment of metals [52]. Therefore, careful consideration should be given to the interpretation of findings based on metals measured in different matrices, as they reflect variable attempts at validation and different windows of exposure (from days to years) [52]. Furthermore, specific metals are subject to differential homeostatic controls that affect how tightly they are regulated by the body and how they are excreted [52]. Finally, element biomarkers in urine should be corrected for the level of urine dilution, which is typically done through adjustment for specific gravity or creatinine. This adjustment was not universally performed in the studies under review.

### Nutritional Status and Dietary Factors

Several studies in the review assessed some aspects of maternal or child diet, such as intakes of foods often known to be sources of metals, supplement use, or the level of essential nutrients measured in urine or whole blood. However, few studies included dietary intakes in statistical models. The assessment of overall nutrient intakes, diet patterns or diet quality may be more informative than focusing on single food groups and may yield important insights into the effect modification of metal toxicity by dietary factors. Among studies included in this scoping review, interactive effects by dietary/nutrient intakes were only investigated in 3 studies on prenatal growth [23–25] and 3 studies on postnatal growth [38, 43, 48]. Additional research on both nutrients and diet pattens/quality within studies on metal exposures is warranted given that nutritional factors play a key role in child growth [1].

Data on micronutrient/supplement intake were collected in 6 studies on postnatal and 9 studies on prenatal growth. Micronutrients play several important roles in the biochemical pathways of metals in the body. For example, the B-vitamins play a vital role in the methylation of arsenic [53, 54]. Iron and calcium have been shown to counteract the absorption of lead in the gastrointestinal tract. Iron and lead are both shuttled by the divalent metal transporter 1 (DMT1) across apical cell membranes in the small intestine; low iron intakes may result in increased absorption of lead [55, 56]. Iron and cadmium compete for DMT1 for absorption in the intestines, and the expression of these transporters is influenced by essential minerals such as zinc [57]. Beyond arsenic, lead, and cadmium, the role of micronutrients in the metabolism of toxic elements remains unclear, thereby highlighting the need for more research.

The levels of essential minerals such as iron, selenium, manganese, and zinc were included in several studies and commonly measured in urine or whole blood. These biomarkers need to be interpreted with caution; their inclusion in models to represent nutritional status may be inappropriate [58]. For example, selenium, manganese or zinc in urine are not considered to be acceptable biomarkers of nutritional status in the human body [59]. Caution needs to be exercised even for validated and accepted biomarkers of nutritional status like serum ferritin. This biomarker, which reflects circulating iron stores, is affected by infection and inflammation, necessitating the inclusion of additional markers such as C-reactive protein, to appropriately classify individuals as iron deficient or sufficient.

One of the strongest determinants of fetal growth is maternal nutritional status and the supply of nutrients to the fetus throughout gestation [60]. It is noteworthy that most studies in this review did account for maternal nutritional status, typically BMI. However, few studies utilized indicators reflective of the mother’s iron status such as maternal hemoglobin or anemia. Maternal anemia is common, with a prevalence of 11.4% among U.S. women participating in the Women, Infants and Children (WIC) program [61], and an overall global prevalence of 36.8% [62]. There is strong evidence that maternal anemia is associated with the risk of LBW [63], suggesting that hemoglobin should be included in statistical models. Furthermore, there is evidence that iron deficiency and/or anemia may co-occur to various extents with overweight/obesity in women of reproductive age across many sociodemographic settings [64]. Therefore, it may be insufficient to account for BMI as the sole indicator of maternal nutritional status. Hemoglobin is relatively easy to assess in field settings with portable hemoglobinometers, but it must be noted that it is not a sensitive indicator of early stages iron deficiency [65].

### Statistical Modeling

Mixture models are gaining in popularity in environmental epidemiology to investigate the joint effects of multiple exposures on health. In this scoping review, 22 studies on prenatal and 5 studies on postnatal growth specifically used mixture models, mainly BKMR or WQS; fewer studies employed quantile g-computation. The comparability across studies utilizing these methods is limited because of unique strengths and limitations of each study, due to large differences in the number and type of metals they included in the mixture, and the severity of exposures. Furthermore, the window of exposure reflected by the biomarker (long-, medium or short-term) is important to consider. For example, a mixture containing urinary lead and cadmium is testing the individual and joint effects of short-term lead but long-term cadmium exposure. A better understanding of how the toxic elements affect growth individually and how they interact to affect different biological pathways is needed. Finally, including valid biomarkers of essential elements in the mixture may reveal associations that are contrary to hypotheses for single metals. Considering mixture effects on growth for children below or above specific thresholds that indicate nutritional deficiency or over-nutrition may also be worthwhile.

Some studies performed a selection process on metals by first conducting linear models and then including in mixture modeling only those metals identified by linear models. Although this addresses the need for a more parsimonious set of features in a mixture function, especially for BKMR that tests non-linear relationships, going from a model with linearity assumptions to a model that is free of those assumptions is somewhat counter-intuitive. Having an a priori reason or hypothesis to include metals in mixtures would be one way of addressing these issues. As discussed above, some of the mixture analyses are based on metals measured in urine and others on metals measured in blood or other biological samples that may or may not be valid biomarkers for a given exposure. Therefore, care is needed in making decisions on which metals are selected for mixture modeling and on what basis.

## Conclusions

This scoping review revealed a large body of literature published between years 2010 and 2023 across different settings and focusing on prenatal or postnatal growth outcomes. The studies ranged in the number of metals assessed, but lead, cadmium, mercury and arsenic were most common. The review revealed the following knowledge gaps – (i) Despite the large number of studies that included multiple metals, few studies assessed the effects of metal mixtures in relation to pre- and postnatal growth. More mixture analyses are needed; (ii) Data on dietary or micronutrient intake were collected in several studies but were largely excluded from statistical analyses. Diet is a modifiable determinant of growth in children, which makes it amenable to interventions. Including foods and diets in statistical analyses helps enhance the understanding of the dual role that these factors may play as mitigators of toxicity and sources of metal exposure; (iii) Many studies assessed essential minerals via ICP-MS and some included those in statistical models as part of the mixture. Very few studies measured the levels of validated nutritional status indicators. Given the role that nutrients play in the absorption and metabolism of heavy metals, their inclusion in analyses is essential in future studies by carefully selecting recognized biomarkers of nutritional status.

## Supplementary Information

Below is the link to the electronic supplementary material.

ESM 1 (XLSX 48.9 KB)

## Data Availability

No datasets were generated or analysed during the current study.

## References

[1] Grasgruber P, Hrazdíra E. Nutritional and socio-economic predictors of adult height in 152 world populations. Econ Hum Biol. 2020;37:100848. 10.1016/j.ehb.2020.100848 32247188

[2] Jamaluddine Z, et al. Effects of size at birth on health, growth and developmental outcomes in children up to age 18: an umbrella review. Arch Dis Child. 2023;108(12):956–69. 37339859 10.1136/archdischild-2022-324884 PMC11474254

[3] Gluckman PD, et al. Effect of in utero and early-life conditions on adult health and disease. N Engl J Med. 2008;359(1):61–73. 18596274 10.1056/NEJMra0708473 PMC3923653

[4] de Onis M, Branca F. Childhood stunting: a global perspective. Matern Child Nutr. 2016;12(1):12–26. 27187907 10.1111/mcn.12231 PMC5084763

[5] Zhang Y, et al. Association of large for gestational age with cardiovascular metabolic risks: a systematic review and meta-analysis. Obesity. 2023;31(5):1255–69. 37140379 10.1002/oby.23701

[6] Sahoo K, et al. Childhood obesity: causes and consequences. J Family Med Prim Care. 2015;4(2):187–92. 25949965 10.4103/2249-4863.154628 PMC4408699

[7] Witkowska D, Słowik J, Chilicka K. Heavy metals and human health: possible exposure pathways and the competition for protein binding sites. Molecules. 2021;26(19):6060. 10.3390/molecules26196060. 10.3390/molecules26196060 PMC8511997 34641604

[8] Dack K, et al. Mercury and prenatal growth: a systematic review. Int J Environ Res Public Health. 2021;18(13):7140. 10.3390/ijerph18137140. 34281082 10.3390/ijerph18137140 PMC8297189

[9] Rahman A, Granberg C, Persson L. Early life arsenic exposure, infant and child growth, and morbidity: a systematic review. Arch Toxicol. 2017;91(11):3459–67. 28905217 10.1007/s00204-017-2061-3

[10] Khoshhali M, et al. Maternal exposure to cadmium and fetal growth: a systematic review and meta-analysis. Biol Trace Elem Res. 2020;195(1):9–19. 31401745 10.1007/s12011-019-01819-y

[11] Vigeh M, Sahebi L, Yokoyama K. Prenatal blood lead levels and birth weight: a Meta-analysis study. J Environ Health Sci Eng. 2023;21(1):1–10. 37155699 10.1007/s40201-022-00843-w PMC10163201

[12] Dettwiler M, Flynn AC, Rigutto-Farebrother J. Effects of non-essential toxic trace elements on pregnancy outcomes: a narrative overview of recent literature syntheses. Int J Environ Res Public Health. 2023;20(13):6211. 10.3390/ijerph20085536. 37107818 10.3390/ijerph20085536 PMC10139051

[13] Ashrap P, et al. Maternal blood metal and metalloid concentrations in association with birth outcomes in Northern Puerto Rico. Environ Int. 2020;138. 10.1016/j.envint.2020.105606 PMC7198231 32179314

[14] Issah I, et al. Exposure to metal mixtures and adverse pregnancy and birth outcomes: a systematic review. Sci Total Environ. 2024;908:168380. 10.1016/j.scitotenv.2023.168380 37963536

[15] Page MJ, et al. The PRISMA 2020 statement: an updated guideline for reporting systematic reviews. BMJ. 2021;372:n71. 33782057 10.1136/bmj.n71 PMC8005924

[16] Bah HAF, et al. Maternal exposure to potentially toxic metals and birth weight: preliminary results from the DSAN-12 M birth cohort in the recôncavo baiano, Brazil. Int J Environ Res Public Health. 2023;20(13):6211. 10.3390/ijerph20136211. 10.3390/ijerph20136211 PMC10340643 37444059

[17] Chen Z, et al. Placental transfer and concentrations of cadmium, mercury, lead, and selenium in mothers, newborns, and young children. J Expo Sci Environ Epidemiol. 2014;24(5):537–44. 24756102 10.1038/jes.2014.26 PMC4329243

[18] Deyssenroth MA, et al. Intrauterine multi-metal exposure is associated with reduced fetal growth through modulation of the placental gene network. Environ Int. 2018;120:373–81. 30125854 10.1016/j.envint.2018.08.010 PMC6288802

[19] Gundacker C, et al. Perinatal lead and mercury exposure in Austria. Sci Total Environ. 2010;408(23):5744–9. 10.1016/j.scitotenv.2010.07.079 20825977

[20] Signes-Pastor AJ, et al. Prenatal exposure to metal mixture and sex-specific birth outcomes in the new Hampshire birth cohort study. Environ Epidemiol. 2019;3(5):e068. 10.1097/EE9.0000000000000068. 10.1097/EE9.0000000000000068 PMC6914313 31844832

[21] Yim G, et al. Metals and per- and polyfluoroalkyl substances mixtures and birth outcomes in the new Hampshire birth cohort study: beyond single-class mixture approaches. Chemosphere. 2023;329:138644. 37031836 10.1016/j.chemosphere.2023.138644 PMC10208216

[22] Lazarevic N, et al. Prenatal exposure to mixtures of persistent environmental chemicals and fetal growth outcomes in Western Australia. Int J Hyg Environ Health. 2022;240:113899. 34883336 10.1016/j.ijheh.2021.113899

[23] Jung CR, et al. Exposure to heavy metals modifies optimal gestational weight gain: a large nationally representative cohort of the Japan environment and children’s study. Environ Int. 2021;146:106276. 33264735 10.1016/j.envint.2020.106276

[24] Luo Y, et al. Maternal blood cadmium, lead and arsenic levels, nutrient combinations, and offspring birthweight. BMC Public Health. 2017;17(1):354. 28438148 10.1186/s12889-017-4225-8 PMC5402649

[25] Zhang X, et al. Joint associations among prenatal metal mixtures and nutritional factors on birth weight z-score: evidence from an urban U.S. population. Environ Res. 2022;208:112675. 10.1016/j.envres.2022.112675. 34995543 10.1016/j.envres.2022.112675 PMC8916990

[26] García-Villarino M, et al. Exposure to metal mixture and growth indicators at 4–5 years. A study in the INMA-Asturias cohort. Environ Res. 2022;204(Pt D):112375–p. 10.1016/j.envres.2021.112375 PMC8671344 34785205

[27] Huang W, et al. In-utero co-exposure to toxic metals and micronutrients on childhood risk of overweight or obesity: new insight on micronutrients counteracting toxic metals. Int J Obes (Lond). 2022;46(8):1435–45. 35589962 10.1038/s41366-022-01127-x PMC9329205

[28] Kupsco A, et al. Prenatal metal concentrations and childhood cardiometabolic risk using bayesian kernel machine regression to assess mixture and interaction effects. Epidemiology. 2019;30(2):263–73. 10.1097/EDE.0000000000000962 PMC6402346 30720588

[29] Signes-Pastor AJ, et al. Exposure to a mixture of metals and growth indicators in 6-11-year-old children from the 2013-16 NHANES. Expo Health. 2021;13(2):173–84. 10.1007/s12403-020-00371-8 PMC8210664 34151044

[30] Moody EC, et al. Environmental exposure to metal mixtures and linear growth in healthy Ugandan children. PLoS ONE. 2020;15(5):e0233108. 32413070 10.1371/journal.pone.0233108 PMC7228047

[31] Choi J, et al. Low-level toxic metal exposure in healthy weaning-age infants: association with growth, dietary intake, and iron deficiency. Int J Environ Res Public Health. 2017;14(4):388. 10.3390/ijerph14040388. 28383506 10.3390/ijerph14040388 PMC5409589

[32] Jedrychowski WA, et al. Depressed height gain of children associated with intrauterine exposure to polycyclic aromatic hydrocarbons (PAH) and heavy metals: the cohort prospective study. Environ Res. 2015;136:141–7. 25460630 10.1016/j.envres.2014.08.047 PMC4262637

[33] Orun E, Yalcin S. Lead, mercury, cadmium levels in breast milk and infant hair in the late period of lactation from Ankara, Turkey. Arch Dis Child. 2014;99:A367–8. 10.1080/09603123.2021.1929872 34092151

[34] Zeng X, et al. Heavy metal exposure has adverse effects on the growth and development of preschool children. Environ Geochem Health. 2019;41(1):309–21. 29696494 10.1007/s10653-018-0114-z

[35] Nasab H, et al. Association of As, Pb, Cr, and Zn urinary heavy metals levels with predictive indicators of cardiovascular disease and obesity in children and adolescents. Chemosphere. 2022;294:133664. 10.1016/j.chemosphere.2022.133664 35066075

[36] Vrijheid M, et al. Early-Life environmental exposures and childhood obesity: an Exposome-Wide approach. Environ Health Perspect. 2020;128(6):67009. 32579081 10.1289/EHP5975 PMC7313401

[37] Dhooge W, et al. Internal exposure to pollutants and body size in Flemish adolescents and adults: associations and dose-response relationships. Environ Int. 2010;36(4):330–7. 10.1016/j.envint.2010.01.005 20181395

[38] Egwunye J, et al. The role of fingernail selenium in the association between arsenic, lead and mercury and child development in rural Vietnam: a cross-sectional analysis. Br J Nutr. 2023;129(9):1589–97. 35535482 10.1017/S0007114522001374

[39] Gardner RM, et al. Environmental exposure to metals and children’s growth to age 5 years: a prospective cohort study. Am J Epidemiol. 2013;177(12):1356–67. 23676282 10.1093/aje/kws437 PMC3676155

[40] Haddad N, et al. An exposome-wide association study on body mass index in adolescents using the National health and nutrition examination survey (NHANES) 2003–2004 and 2013–2014 data. Sci Rep. 2022;12(1):8856. 35614137 10.1038/s41598-022-12459-z PMC9132896

[41] Yang H, et al. Effects of lead and cadmium exposure from electronic waste on child physical growth. Environ Sci Pollut Res Int. 2013;20(7):4441–7. 23247522 10.1007/s11356-012-1366-2

[42] Ashley-Martin J, et al. Blood metal levels and early childhood anthropometric measures in a cohort of Canadian children. Environ Res. 2019;179(Pt A):108736. 31541908 10.1016/j.envres.2019.108736

[43] Fábelová L, et al. Hair concentration of trace elements and growth in homeless children aged < 6years: results from the ENFAMS study. Environ Int. 2018;114:318–25. 29150339 10.1016/j.envint.2017.10.012

[44] Fan Y, Zhang C, Bu J. Relationship between selected serum metallic elements and obesity in children and adolescent in the U.*S.* nutrients. 2017;9(2):104. 10.3390/nu9020104 PMC5331535 28165362

[45] Li M, et al. Association between heavy metals exposure and height in Chinese preschoolers. J Occup Environ Med. 2023;65(7):567–72. 37171101 10.1097/JOM.0000000000002834

[46] Shan Q. Trend analysis of the association of urinary metals and obesity in children and adolescents. Chemosphere. 2022;307(Pt 1):135617. 35820478 10.1016/j.chemosphere.2022.135617

[47] Shao W, et al. Association between level of urinary trace heavy metals and obesity among children aged 6–19 years: NHANES 1999–2011. Environ Sci Pollut Res Int. 2017;24(12):11573–81. 28321702 10.1007/s11356-017-8803-1

[48] Donangelo CM, et al. Lead exposure and indices of height and weight in Uruguayan urban school children, considering co-exposure to cadmium and arsenic, sex, iron status and dairy intake. Environ Res. 2021;195:110799. 33508259 10.1016/j.envres.2021.110799 PMC10916356

[49] Aguree S, Gernand AD. Plasma volume expansion across healthy pregnancy: a systematic review and meta-analysis of longitudinal studies. BMC Pregnancy Childbirth. 2019;19(1):508. 31856759 10.1186/s12884-019-2619-6 PMC6924087

[50] Cheng L, et al. Critical windows of prenatal exposure to cadmium and size at birth. Int J Environ Res Public Health. 2017;14(1):58. 10.3390/ijerph14010058. 28075368 10.3390/ijerph14010058 PMC5295309

[51] Chen W, et al. Progress in ICP-MS analysis of minerals and heavy metals in traditional medicine. Front Pharmacol. 2022;13:891273. 35837276 10.3389/fphar.2022.891273 PMC9274010

[52] Martinez-Morata I, et al. A state-of-the-science review on metal biomarkers. Curr Environ Health Rep. 2023;10(3):215–49. 37337116 10.1007/s40572-023-00402-x PMC10822714

[53] Hall MN, et al. Folate, Cobalamin, Cysteine, Homocysteine, and arsenic metabolism among children in Bangladesh. Environ Health Perspect. 2009;117(5):825–31. 10.1289/ehp.0800164 PMC2685848 19479028

[54] Selhub J. Folate, vitamin B12 and vitamin B6 and one carbon metabolism. J Nutr Health Aging. 2002;6(1):39–42. 11813080

[55] Kordas K. The lead diet: can dietary approaches prevent or treat lead exposure? J Pediatr. 2017;185:224–e2311. 28283259 10.1016/j.jpeds.2017.01.069

[56] Słota M, et al. Relationship between lead absorption and iron status and its association with oxidative stress markers in lead-exposed workers. J Trace Elem Med Biol. 2021;68:126841. 10.1016/j.jtemb.2021.126841 34438315

[57] Zhai Q, Narbad A, Chen W. Dietary strategies for the treatment of cadmium and lead toxicity. Nutrients. 2015;7(1):552–71. 25594439 10.3390/nu7010552 PMC4303853

[58] Höller U, et al. Micronutrient status assessment in humans: current methods of analysis and future trends. TrAC Trends Anal Chem. 2018;102:110–22.

[59] Pikounis TD, et al. Urinary biomarkers of exposure to toxic and essential elements: a comparison of infants fed with human milk or formula. Environ Epidemiol. 2024;8(1):e286. 38343736 10.1097/EE9.0000000000000286 PMC10852378

[60] Wu G, et al. Maternal nutrition and fetal development. J Nutr. 2004;134(9):2169–72. 15333699 10.1093/jn/134.9.2169

[61] Kanu FA, et al. Anemia among pregnant women participating in the special supplemental nutrition program for women, infants, and children - United States, 2008–2018. MMWR Morb Mortal Wkly Rep. 2022;71(25):813–9. 35737575 10.15585/mmwr.mm7125a1

[62] Karami M, et al. Global prevalence of anemia in pregnant women: a comprehensive systematic review and meta-analysis. Matern Child Health J. 2022;26(7):1473–87. 35608810 10.1007/s10995-022-03450-1

[63] Figueiredo A, et al. Maternal anemia and low birth weight: a systematic review and meta-analysis. Nutrients. 2018;10(5):601. 10.3390/nu10050601. 29757207 10.3390/nu10050601 PMC5986481

[64] Irache A, Gill P, Caleyachetty R. The co-occurrence of overweight/obesity and anaemia among adult women, adolescent girls and children living in fifty-two low- and middle-income countries. Public Health Nutr. 2022;25(6):1595–606. 34103123 10.1017/S1368980021002512 PMC9991775

[65] Hanif N, Anwer F. Chronic iron deficiency. In: StatPearls. StatPearls Publishing Copyright © 2024, StatPearls publishing LLC: Treasure Island (FL); 2024. 32809711

